# The effects of yoga exercise on stress relief capacity and emotional changes: a systematic review and meta-analysis

**DOI:** 10.3389/fpsyg.2026.1707131

**Published:** 2026-02-26

**Authors:** Xiaokun Mu, Kai Xu, Xiaolin Wang, Yi Sun, Dengtai Wen, Delong Dong

**Affiliations:** 1 School of Physical Education, Ludong University, Yantai, China; 2 School of Athletic Performance, Shanghai University of Sport, Shanghai, China

**Keywords:** anxiety, depression, meta, stress, yoga

## Abstract

**Background:**

Emotional stability is a central pillar of mental health, and prolonged exposure to negative emotions (depression, anxiety) and stress can lead to impaired social functioning and disruptions in the emotion regulation system, which in turn affects psychological well-being.

**Objective:**

The purpose of this study is to examine the combined benefits of yoga practice for emotional regulation and stress reduction through meta-analysis, while exploring the effects of various moderating factors.

**Methods:**

This study was searched through Pubmed, Embase, Ovid MEDLINE and Cochrane library databases in January 2026. Randomized controlled studies using yoga as an intervention and anxiety, depression, and stress as indicators were included. Risk of bias was reported faithfully according to the Cochrane risk of bias rating requirements and labeled with the GRADE system evaluation level of evidence. Random-effects models were employed to perform effect size (ES) pooling, examine publication bias, and conduct subgroup analyses and regression analyses.

**Results:**

Included 30 controlled experimental studies involving 2,288 participants (age: 13 to 82 years). Compared with control groups, yoga interventions improved stress (ES = −0.54, Low-level Evidence), anxiety (ES = −0.52, Low-level Evidence), and depression (ES = −0.50, Low-level Evidence). Subgroup analyses and regression analyses indicated that age was a significant moderator of stress levels.

**Conclusion:**

Yoga practice has been proven effective in reducing stress and alleviating symptoms of depression and anxiety. As age increases, yoga interventions yield greater effectiveness in stress reduction. In the future, yoga should be considered as a complementary therapy for promoting mental health.

**Systematic review registration:**

PROSPERO, identifier (CRD420251044568).

## Introduction

The World Health Organization (WHO) (World Health Organization, 2022) states in its report that psychological well-being is the foundation for achieving a fulfilling life, and that mental health is influenced by multiple dimensions including both negative and positive emotions (Guney et al., 2010). Among these, depression, anxiety, and stress are the most common mental health issues (Rao and Ramesh, 2015). The American Psychiatric Association has defined depression (Torres, 2020) as a serious mental illness that disrupts a person’s thoughts and behavior while indirectly causing damage to their physical functions (Malik et al., 2017). It manifests as sadness, distress, and emptiness. Stress is an adaptive response generated by the human body when confronting challenges from internal and external environments. It is triggered when external demands exceed an individual’s acceptable threshold, causing psychological and physiological fluctuations (Watode et al., 2015). Prolonged stress induces anxiety in the human body, which is also regarded as an emotional state (Latif et al., 2015), manifesting as excessive worry, physical discomfort, and nervous tension.

Yoga is a form of physical and mental exercise (Rogers and MacDonald, 2015) originating in ancient India (Wahbeh et al., 2008). It is a comprehensive practice combining postures, breath control, and concentration (Mooventhan and Khode, 2014). Modern yoga has evolved with the continuous expansion of asana varieties, giving rise to distinct practice styles. Due to its ability to regulate the nervous system (Sullivan et al., 2018; Bhargav et al., 2021) for mental health benefits, low cost, and broad inclusivity, yoga has become a globally popular activity (Zhang et al., 2021; Cartwright et al., 2020) and has been integrated into healthcare systems as a rehabilitative approach for promoting wellness and disease management.

Multiple meta-analyses have now confirmed that yoga practice can effectively alleviate stress and improve symptoms of depression and anxiety (Değirmenci et al., 2025; Meliani et al., 2025; Neto et al., 2024; Arya et al., 2025; Ding et al., 2024). However, certain limitations remain. First, most meta-analyses focus on patients with existing conditions, neglecting studies on healthy populations. This overlooks yoga’s preventive role in addressing emotional issues and stress reduction. Secondly, some meta-analyses exhibit flaws in the reliability of their results and research methodologies. Given that most controlled trials using yoga as an intervention have limited sample sizes, it is necessary to consider the potential for small-sample studies to overestimate outcomes. Furthermore, a significant number of meta-analyses failed to employ regression models (Thompson and Higgins, 2002) to explore the linear relationships between indicators and their moderating factors, This prevents us from gaining a deeper understanding of which populations and under what conditions yoga interventions are most effective. Consequently, even though yoga practice is known to confer mental health benefits, it remains unable to provide policymakers with reliable clinical recommendations.

The purpose of this study is to examine the combined benefits of yoga practice for emotional regulation and stress reduction through meta-analysis, while exploring the effects of various moderating factors. We will explore the relationship between multiple moderating factors in yoga interventions and psychological indicators through regression models, aiming to provide more precise and reliable guidance for future research and the development of clinical protocols.

## Methods

This systematic evaluation and meta-analysis were conducted in accordance with the Cochrane Guidelines, and the article is based on the normative implementation of Meta-analysis according to the PRISMA statement published in 2020 (Page et al., 2021). This study has been registered with the International Prospective Registry for Systematic Reviews (PROSPERO) under the registration number [CRD420251044568].

### Search strategy

**Figure 1:**
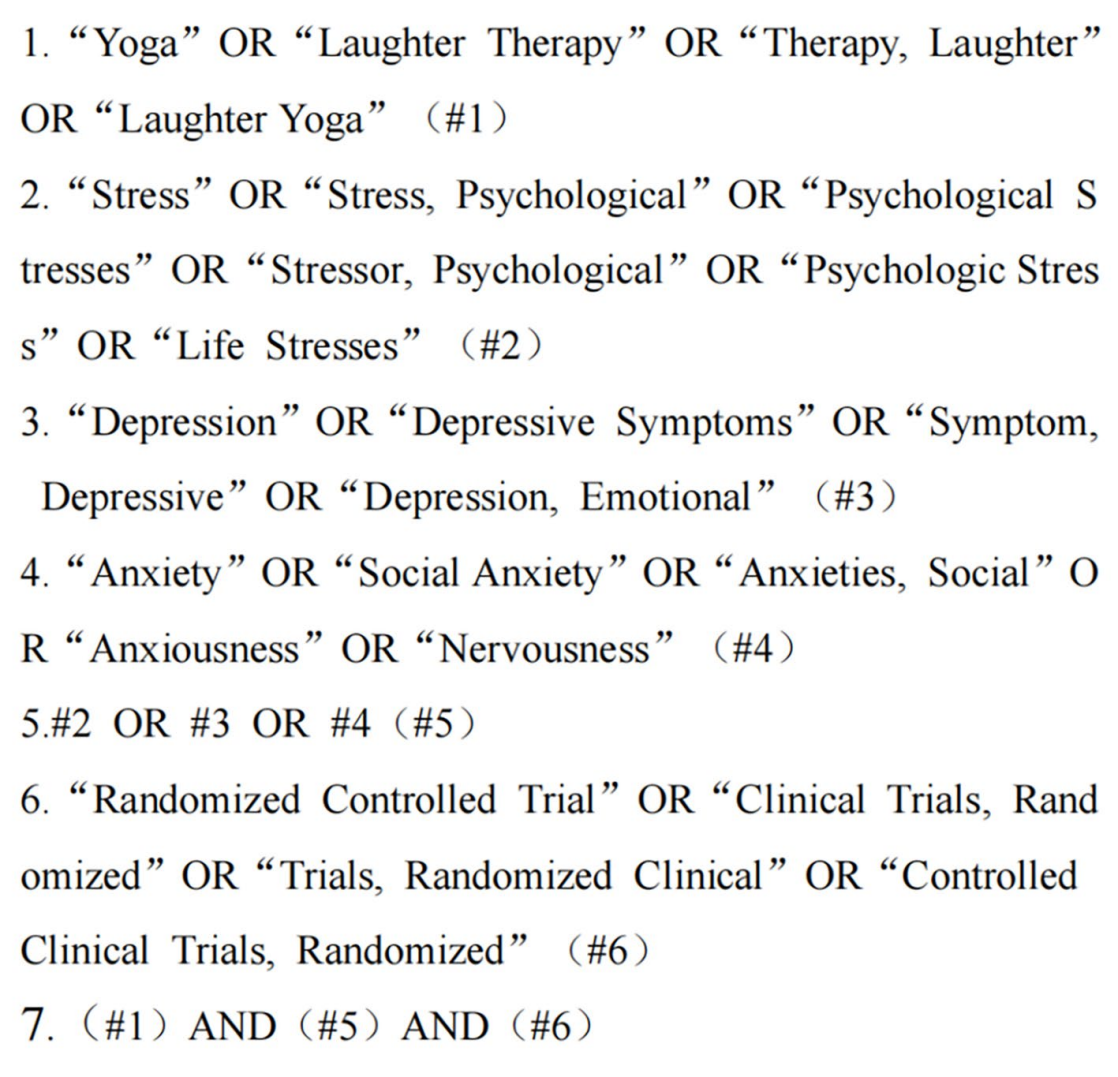
Literature search strategies for PubMed databases.

This study was conducted through a systematic search using Pubmed, Embase, Cochrane library and Ovid medline medical databases. Based on the PICO framework (Population, Intervention, Comparison, Outcome) divided into Population (“Adolescents,” “Adults,” “Older Adults”), Intervention (“Yoga exercise”), comparisons (“Control group,” “Active Control Group”, etc.) and outcomes (depression, stress, pressure). The search for this study had no restrictions on publication date, and the literature was first filtered by browsing through the title and abstracts, eventually transitioning to full-text reading. Take the Pubmed database search strategy as an example (Figure 1).

#### Selection process

Literature was managed using EndNote software (Version X9), where the retrieved literature was first de-duplicated, and based on this the literature was initially screened by reading the titles and abstracts of the literature according to the inclusion and exclusion criteria. Literature screening was conducted independently by two screeners (X.K.M. and K.X.), and if disagreements arose during the review of the readings, a meeting was held to reach a consensus. If consensus could not be reached, a third researcher (X.L.W.) intervened to make the final decision as to whether the criteria for inclusion in the literature were met. At the full-text review stage, the two screeners also worked independently and dealt with disagreements in a manner consistent with the methodology described above.

#### Eligibility criteria

Inclusion and exclusion criteria were designed based on the PICOS framework, and the inclusion criteria were as follows: (1) Participants included healthy adolescents (<18 years), adults (young adults: 18–44 years; middle-aged adults: 45–64 years), and older adults (≥65 years). (2) The intervention group consisted of yoga exercises of varying types and durations; The control groups included a waiting control group, an active control group, and a standard control group, This approach was adopted to avoid overestimating the intervention’s effectiveness. (3) Outcome measures should include at least one emotional and psychological indicator, such as stress, depression, or anxiety. (4) The study design must be a randomized controlled trial.

The exclusion criteria were as follows: (1) Qualitative research, systematic review, research protocol, Graduation Thesis, grey literature, and abstract. (2) Research where stable process control cannot be achieved during experimentation. (3) Excluded subjects who were identified in the report as having unhealthy conditions or severe physiological and psychological disorders.

#### Data collection and conversion process

Data extraction was carried out independently by the second author (K.X.) and the third author (X.L.W.), filling in the information in a pre-designed Microsoft Excel data sheet. When it appeared that a study was missing the data needed for this paper, the first author asked the corresponding author of the study directly, by e-mail, for the information needed. If the authors did not respond then this article will not participate in the merging of the meta-analysis.

#### Data item

This study extracted the required variable data from relevant literature and conducted an analysis. The content included: subject characteristics (subject type, sample size, gender ratio, age range, supervision format), yoga intervention protocols (intervention cycle, intervention frequency, duration per session), and measurement results of emotional and psychological indicators (anxiety, depression, stress).

In subgroup analyses, we coded the following moderator variables:

1. Gender (Mixed, Female)
2. Control type (Active Control Group, Control Group, Wait-list Group)
3. Age (Under 30 years old, Over 30 years old)
4. Intervention Cycle (8 weeks and under, more than 8 weeks)
5. Intervention frequency (3 times or fewer, more than 3 times)
6. Intervention time (Over 50 min, 50 min or less)
7. Training Monitoring (Online, Offline, Offline + Online)

#### Study risk of bias assessment

Risk of bias was assessed independently by the second author (K.X.) and the third author (X.L.W.), consistent with the literature screening process. In the event of a dispute, the issue was resolved through discussion; if it could not be resolved, the fourth author (Y.S.) still intervened to adjudicate. During this process, the risk of bias assessment tool, Risk of bias 2, was used to evaluate the following domains: whether the randomization process was appropriate, Deviation from the established intervention or not, Were the outcome data complete, Were outcome measures objective, Was there selective reporting, The above five categories were assessed (Higgins et al., 2011).

#### Data synthesis and effect measures

Statistical analysis of the articles was performed using the metafor package in R software (version 4.4.2). This meta-analysis employed a random-effects model and integrated effect sizes using inverse variance weighting. When calculating the heterogeneity variance *τ^2^*, the restricted maximum likelihood (REML) method was selected. The final pooled effect size and corresponding confidence intervals were calculated based on the *τ^2^* value estimated using the REML method.

Given that outcome measures involved multiple assessment tools, the standardized mean difference (SMD) (Nagashima et al., 2019) was prioritized for calculation of the pooled main effect. SMDs and their 95% confidence intervals were extracted from individual studies. Given the small sample sizes in the included studies, Hedges and Olkin’s g-corrected effect size was employed and converted using the following formula (Hedges and Olkin, 2014).

$$\mathrm{ES}=\frac{\left({\bar{X}}_{1}-{\bar{X}}_{2}\right)}{SD_{\text{pooled}}}\times (1-\frac{3}{4(n_{1}+n_{2}-2)-1})$$

X_1_ and X_2_ represent the post-experiment means for the experimental and control groups, respectively, while n_1_ and n_2_ denote the sample sizes for the experimental and control groups after the experiment. SD-pooled denotes the pooled standard deviation for the experimental and control groups, calculated using the following formula (Hedges and Olkin, 2014):

$$SD_{\text{pooled}}=\sqrt{\frac{\left((n_{1}-1)\times SD_{1}^{2}+(n_{2}-1)\times SD_{2}^{2}\right)}{n_{1}+n_{2}-2}}$$

SD1 and SD2 represent the standard deviations of the experimental group and control group post-experiment, respectively, while n1 and n2 denote the sample sizes of the experimental group and control group post-experiment.

Its magnitude levels can be categorized as follows: (1) < 0.2: Negligible, lacking practical significance. (2) 0.2–0.5: Minor effect. (3) 0.5–0.8: Moderate effect. (4) ≥ 0.8: Substantial effect (Cohen, 2013).

I^2^ is used as the primary basis for assessing heterogeneity, which is categorized by interval (Nakagawa et al., 2017): (1) Low heterogeneity: 0–25%. (2) Moderate heterogeneity: 25–75%. (3) High heterogeneity: >75%.

#### Subgroup and regression analysis

Subgroup analysis and regression analysis were conducted to investigate sources of heterogeneity and influencing factors. Subgroup analysis encompassed seven dimensions: control group type, gender, age, intervention cycle, intervention frequency, intervention duration, and training supervision. Regression analysis was performed across three dimensions: yoga intervention cycle, intervention frequency, and participant age (Binney and Mansournia, 2024). The meta-regression analysis in this study was conducted within a random-effects framework, We evaluated the relationship using both linear models, REML was employed to estimate the heterogeneity variance (*τ^2^*) across studies, Compared to traditional maximum likelihood estimation, REML provides more robust variance component estimates in small-sample scenarios (Rico-González et al., 2022).

#### Publication bias and sensitivity analysis

Publication bias was shown by funnel plot with Egger’s test (Peters et al., 2008; Egger et al., 1997), and the Egger’s test result of *p* > 0.05 was without publication bias, while *p* < 0.05 was expressed as having publication bias.

To assess the robustness of the pooled results, we conducted a sensitivity analysis. First, we employed outlier detection to identify studies with potentially excessive influence on the meta-analysis outcomes (Viechtbauer and Cheung, 2010). Subsequently, using leave-one-out analysis (Patsopoulos et al., 2008), we sequentially excluded each study and repeated the meta-analysis to observe the independent impact of any single study on the overall effect size estimate.

#### Certainty assessment

Levels of evidence were assessed using the GRADE (Grading of Recommendations Assessment, Development, and Evaluation) methodology (Schünemann et al., 2019; Guyatt et al., 2011). with two authors (X.K.M. and K.X.) assessing separately, discussing disagreements when they arose, and, if they could not be resolved a third author (X.L.W.) The third author decided the results. The level of evidence (Guyatt et al., 2011) was categorized from top to bottom as “high, medium, low, and very low”. The assessment of the level of evidence was based on the following criteria:

(1) risk of bias: when the risk of bias of the literature included in the observational indicator was “some concern,” it was lowered by one level; when the risk of bias was “high,” it was lowered by two levels. (2) inconsistency: if the heterogeneity between studies exceeded 25%, it was lowered by one level; if it exceeded 75%, it was lowered by two levels. (3) Imprecision: if the statistical difference is not significant, it will be downgraded by one level. (4) Risk of publication bias: if the result obtained by Eggers’ test shows that the *p* value is less than 0.05, it will be downgraded by one level.

## Results

### Study selection

A search of medical databases (PubMed, Embase, Cochrane Library, and Ovid MEDLINE) yielded 4,426 articles. After screening, we excluded 1,689 duplicate articles. Using the PICO criteria to screen the remaining 2,737 articles, we further excluded 2,648 articles. This screening process was conducted from January 9 to January 11, 2026.

**Figure 2:**
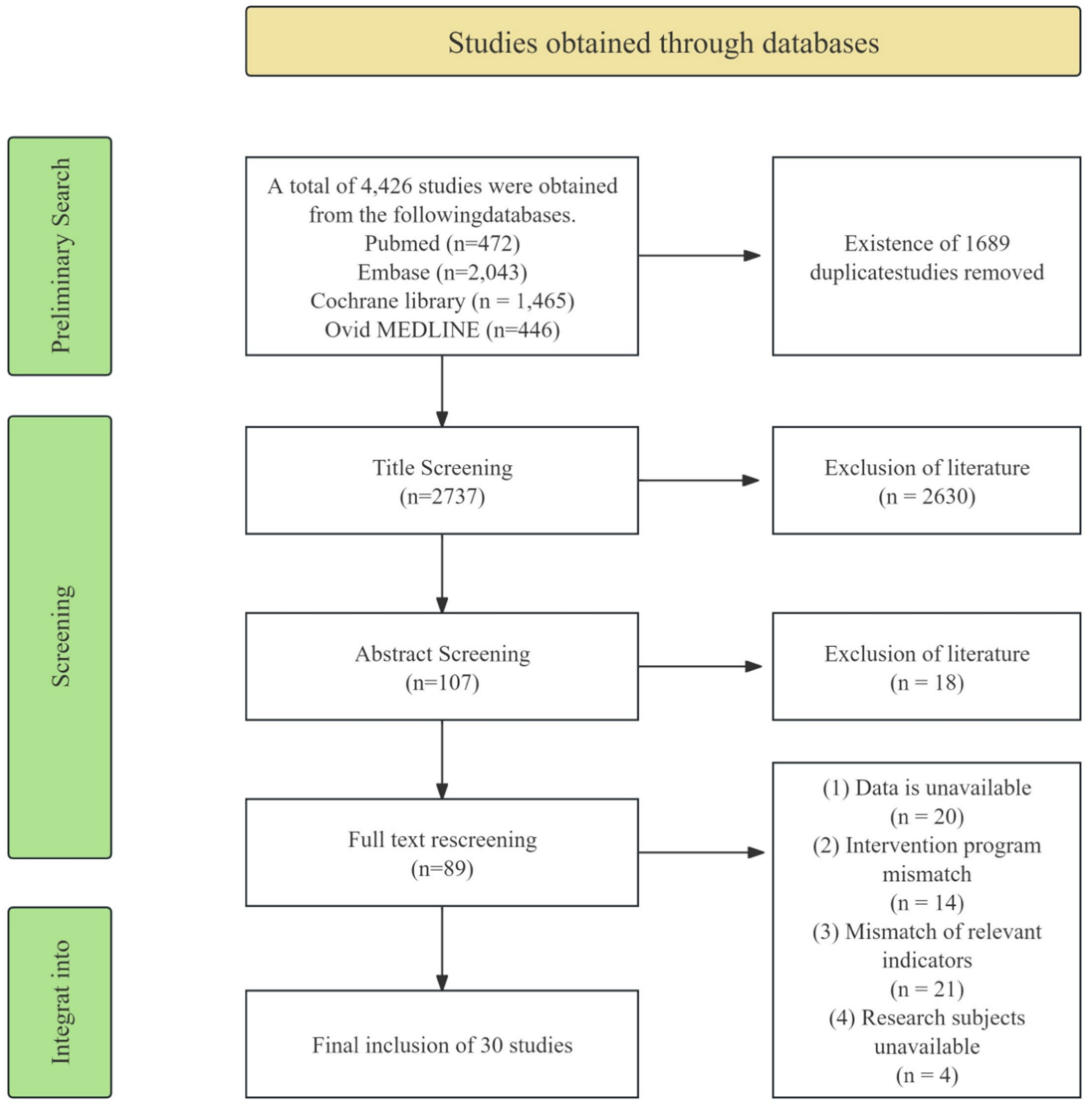
Literature screening flowchart.

Subsequently, we conducted a full-text analysis of 89 studies. Among these, 30 studies met the predefined inclusion criteria and were incorporated into this review. The remaining 59 studies were excluded due to missing data and failure to meet criteria regarding indicators and intervention protocols. The entire analysis phase spanned from January 12 to January 17, 2026. The specific screening process and results are presented in (Figure 2).

### Study characteristics

This review included 30 randomized controlled trials (Brandão et al., 2024; Castellote-Caballero et al., 2024; Chu et al., 2015; Chhugani et al., 2018; Phansikar et al., 2023; Wadhen and Cartwright, 2021; Waechter et al., 2021; Metri et al., 2023; Baklouti et al., 2023; Hewett et al., 2018; Hilcove et al., 2021; Lin et al., 2015; Mandal et al., 2021; Marques et al., 2017; Mathad et al., 2017; Papp et al., 2019; Quach et al., 2016; Yas and Incesu, 2025; Günebakan and Acar, 2023; Cheema et al., 2013; Haag et al., 2024; Kaya et al., 2025; Dol, 2019; Chen et al., 2010; Maddux et al., 2018; Hartfiel et al., 2012; Biman et al., 2021; Saxena et al., 2020; Varambally et al., 2013; Devi and Singh, 2024) involving a total of 2,288 participants (with a higher proportion of women), ranging from 25 to 174 participants per study. Participants’ ages spanned from 13 to 82 years. Yoga styles included Hatha Yoga, Integrated Yoga, and Laughter Yoga. Intervention cycle ranged from 4 to 28 weeks, with frequencies varying from 1 to 6 sessions per week. Each session lasted between 25 and 75 min (Table 1).

**Table 1:** Basic characteristics of the included studies.

Researcher (year)	Participant			Type of intervention				Outcome
	Sample size (M/F)	Age (mean, range)	Subject type	Types of yoga	Intervention period (week)	Intervention frequency (d/wk)	Intervention time (min)	
Brandão et al. (2024)	62(6/56)	21	Student	Kundalini Yoga	6	1	60	①
Castellote-Caballero et al. (2024)	129(63/66)	20	Student	Hatha Yoga	12	2	60	②③
Chu et al. (2015)	52(0/52)	26	Volunteer	Integrated Yoga	8	2	60	②③④
Chhugani et al. (2018)	30(0/30)	20-50	Healthcare workers	Integrated Yoga	4	6	60	①
Phansikar et al. (2023)	86(16/70)	42	Volunteer	Integrated Yoga	8	3	50	②⑤
Wadhen and Cartwright (2021)	34(3/31)	42	Office worker	Hatha Yoga	6	3	50	①
Waechter et al. (2021)	28(13/15)	26	Student	Hatha Yoga	12	2	60	②③④
Metri et al. (2023)	50(0/50)	39	Teacher	Integrated Yoga	6	4	60	①
Baklouti et al. (2023)	160(98/62)	65-80	Volunteer	Hatha Yoga	4	2	45	①
Hewett et al. (2018)	63(13/50)	37	Volunteer	Bikram Yoga	16	5	Not reported	②
Hilcove et al. (2021)	78(4/17)	43	Healthcare workers	Integrated Yoga	6	5	Not reported	②
Lin et al. (2015)	60(12/48)	31	Mental Health Professionals	Integrated Yoga	12	1	60	⑩
Mandal et al. (2021)	110(30/80)	34	Healthcare workers	Integrated Yoga	12	2	50	②
Marques et al. (2017)	25(0/25)	82	Volunteer	Chair-based Yoga	28	3	50	②
Mathad et al. (2017)	80(0/80)	17-30	Student	Integrated Yoga	8	5	60	②
Papp et al. (2019)	50	20–39	Student	Hatha Yoga	6	1	60	②⑤
Quach et al. (2016)	118(49/69)	13	Student	Hatha Yoga	4	2	45	②⑪
Yas and Incesu (2025)	83(12/71)	22	Student	Laughter Yoga	5	1	40	②
Günebakan and Acar (2023)	36(0/36)	28	Volunteer	Hatha Yoga	6	2	45	③⑦
Cheema et al. (2013)	37(7/30)	38	Office worker	Hatha Yoga	10	3	50	③
Haag et al. (2024)	40(5/35)	23	Student	Tantra Yoga	8	2	75	②
Kaya et al. (2025)	90(45/45)	Not reported	Student	Laughter Yoga	4	2	45	②
Dol (2019)	40	Not reported	Student	Yoga nidra	8	2	60	⑥
Chen et al. (2010)	59	Not reported	Volunteer	Silver yoga	12	3	70	⑧
Maddux et al. (2018)	80(14/66)	46	Volunteer	Power Yoga	8	2	60	②⑤
Hartfiel et al. (2012)	59(6/53)	45	Volunteer	Dru Yoga	8	1	50	②
Biman et al. (2021)	155(70/85)	38	Manual workers	Integrated Yoga	12	4	60	②
Saxena et al. (2020)	174(62/112)	15	Student	Hatha Yoga	12	2	25	②
Varambally et al. (2013)	29	18–60	Healthcare workers	Integrated Yoga	12	3	45	⑤
Devi and Singh (2024)	40	18–23	Student	Integrated Yoga	8	5	60	⑨

The scales used in the test are as follows:① DASS: Depression Anxiety Stress Scales; ② PSS: Perceived Stress Scales; ③ STAI: State–Trait Anxiety Inventory; ④ CES-D: Center for Epidemiologic Studies Depression Scale; ⑤ HADS: Hospital Anxiety and Depression Scale; ⑥ VASS: Visual Analog Scale for Stress; ⑦ BDI: Beck Depression Inventory; ⑧ TDQ: Taiwanese Depression Questionnaire; ⑨ SCAT: Sports Competition Anxiety test; ⑩ WRSS: Work Related Stress Scale. ⑪ Screen for Child Anxiety and Related Emotional Disorders.

### Risk of bias in the individual studies

**Figure 3:**
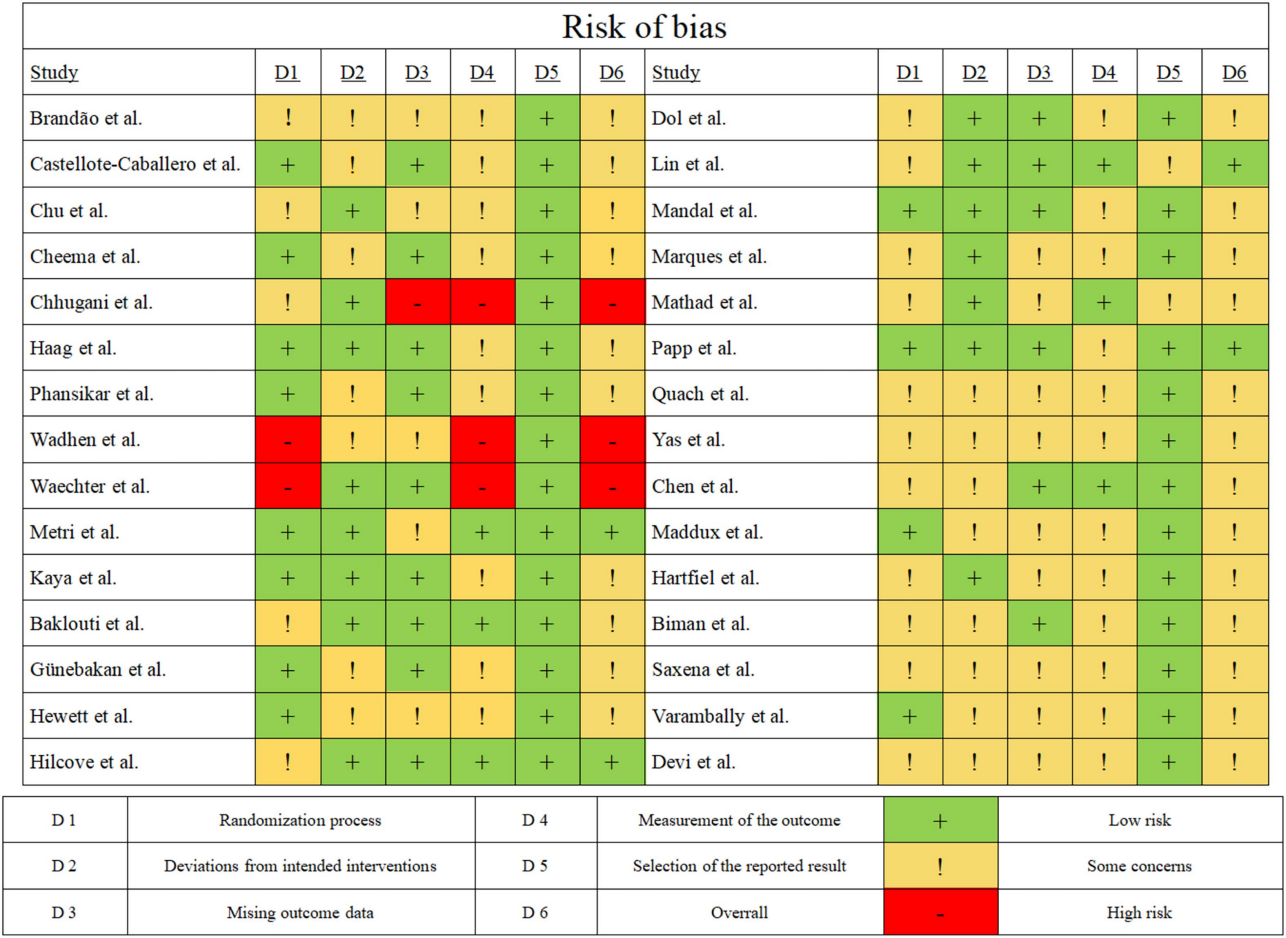
Inclusion of literature risk of bias assessment chart. This figure presents a bubble plot of linear regression analysis for stress indicators, illustrating the association effects of each continuous variable. Each dot in the bubble plot represents an independent effect, with dot size reflecting the weight of the combined main effect. As effect values increase, dot color deepens. The solid black line denotes the regression line; the gray area indicates the 95% confidence interval; the purple area represents the 95% prediction interval. The yellow dashed line marks the −0.5 time period effect. AIC: Akaike Information Criterion. BIC: Bayesian Information Criterion.

Randomized controlled studies were assessed using the Risk of Bias 2 tool, as described in (Figure 3 and Supplementary Figures 1–3). In the analysis of studies related to stress symptoms, 3 out of 25 studies had a high overall risk of bias. In the analysis of studies related to anxiety symptoms, 3 out of 15 studies had a high overall risk of bias. In the analysis of studies related to depressive symptoms, 3 out of 13 studies had a high overall risk of bias.

In the analysis of risk of bias, it was found that the biased results were mainly affected by the randomization process, missing outcome data and bias in outcome measurements. Some of the studies did not provide a detailed explanation of the hidden process of randomization and there were instances of loss of data from the researcher during the experiment and uncertainty about the implementation of blinding of the testers. While these issues are critical to the quality of the study, they are also issues that cannot be completely avoided in randomized controlled trials that conduct interventions.

In terms of reporting results, all 30 studies faithfully reported the results. Overall, the risk of bias indicates that the vast majority of studies exhibit methodological shortcomings, particularly in the areas of randomization, allocation concealment, implementation of blinding, and outcome measurement.

### Main effect

**Figure 4:**
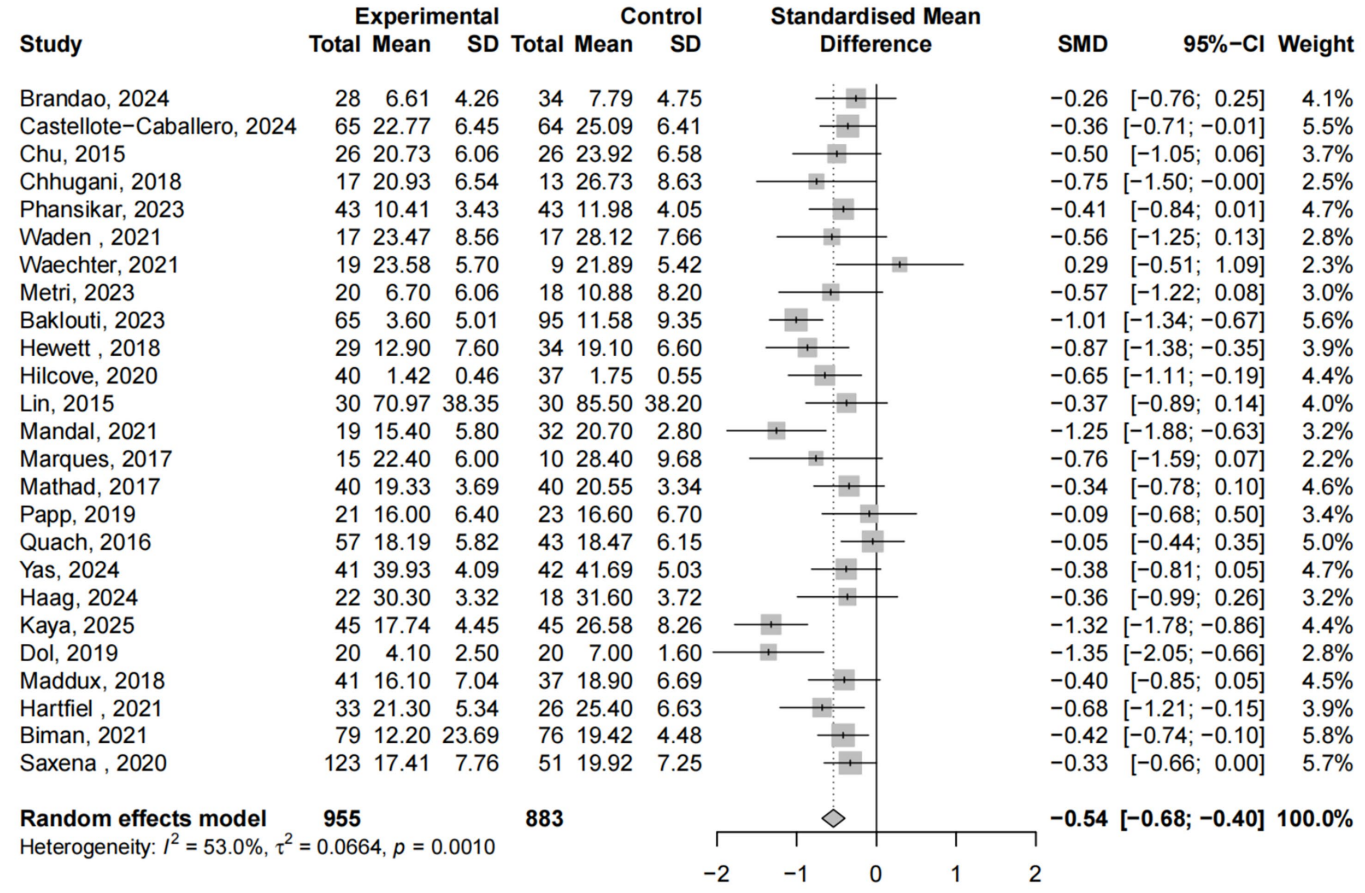
Yoga exercise counteracts stress: forest diagram.

A total of 25 studies involving 1,838 participants examined the effects of yoga interventions on stress reduction. The meta-analysis revealed that yoga interventions effectively alleviated stress symptoms compared to control groups (ES = −0.54, 95% CI: −0.68 to −0.40, *p* < 0.05), with 53% heterogeneity (Figure 4). Subgroup analysis (Supplementary Table 1) revealed that only control group type and age significantly moderated stress changes (*p* < 0.05). Sensitivity analysis excluding Kaya’s study reduced heterogeneity to 40.9% (Supplementary Figure 4).

**Figure 5:**
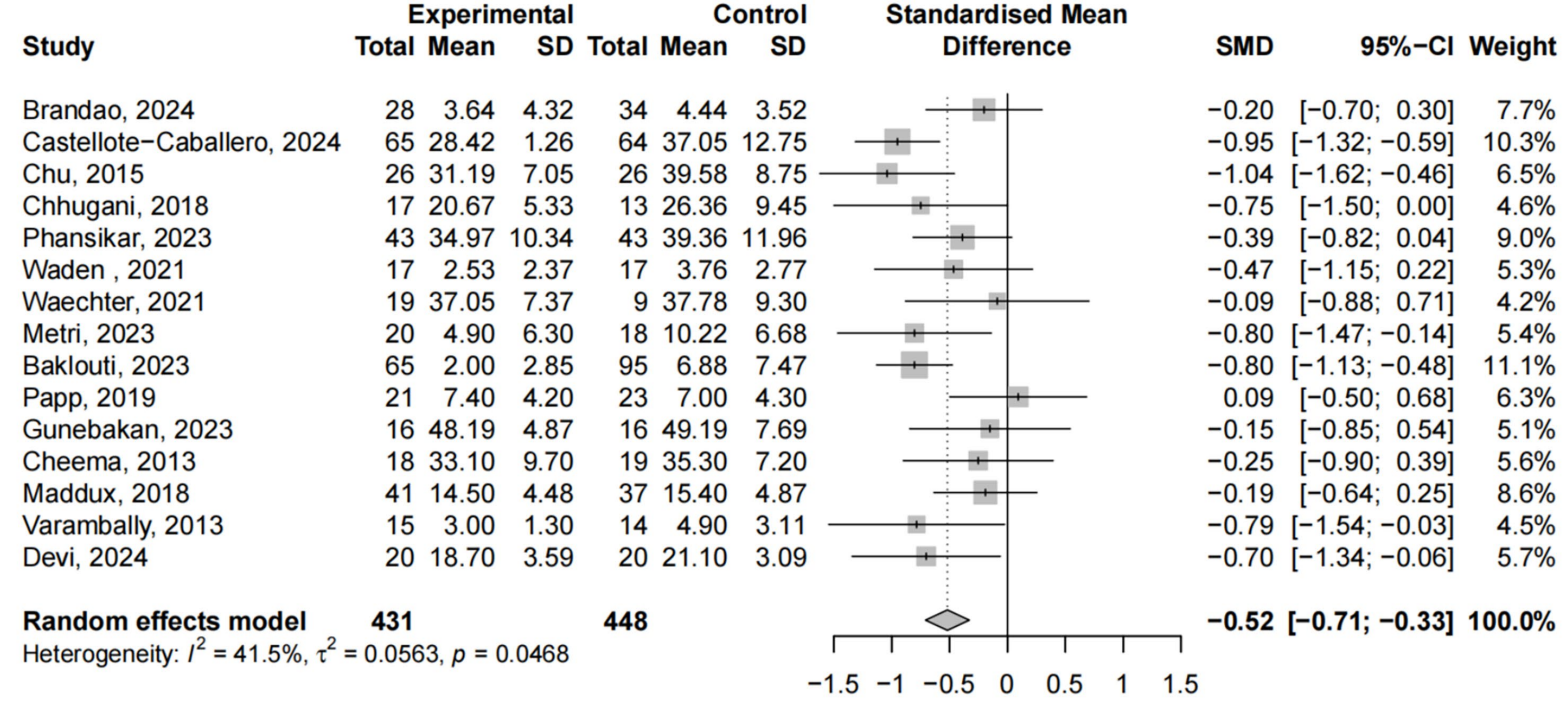
Yoga exercise counteracts anxiety: forest diagram.

A total of 15 studies involving 879 participants examined the effects of yoga interventions on anxiety relief. The meta-analysis revealed that compared to control groups, yoga interventions effectively alleviated stress symptoms (ES = −0.52, 95% CI: −0.71; −0.33, p < 0.05), with 41.5% heterogeneity (Figure 5). Subgroup analysis (Supplementary Table 2) revealed that only the type of control group significantly moderated changes in anxiety (p < 0.05). Sensitivity analysis excluding the Castellote-Caballero’s study reduced heterogeneity to 30.2% (Supplementary Figure 5).

**Figure 6:**
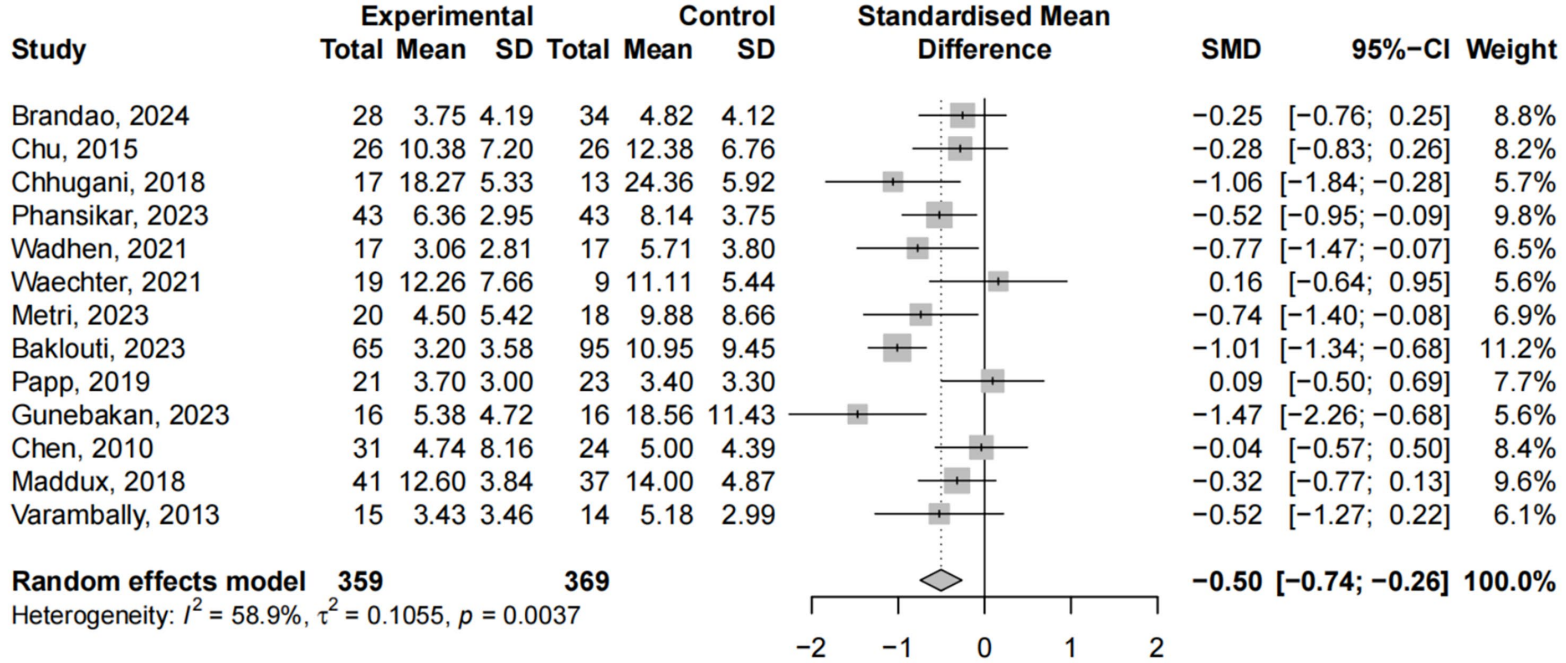
Yoga exercise counteracts depression: forest diagram.

A total of 13 studies involving 728 participants examined the effects of yoga interventions on alleviating depression. The meta-analysis revealed that yoga interventions effectively reduced depressive symptoms compared to control groups (ES = −0.50, 95% CI: −0.74; −0.26, *p* < 0.05), with 58.9% heterogeneity (Figure 6). Subgroup analysis (Supplementary Table 3) revealed that only control group type, intervention cycle, and single-session duration significantly moderated changes in depression (*p* < 0.05). Sensitivity analysis excluding Baklouti’s study reduced heterogeneity to 42.9% (Supplementary Figure 6).

### Meta-regression

**Figure 7:**
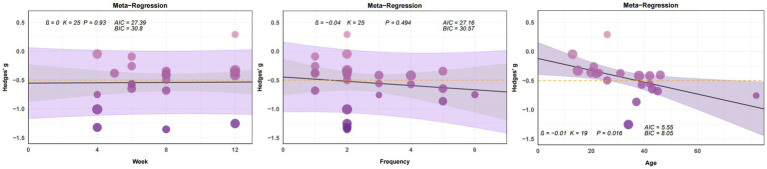
Plot of regression analysis results from the meta-analysis of yoga interventions to cope with stress (week, frequency, age). This figure presents a bubble plot of linear regression analysis for anxiety indicators, illustrating the association effects of each continuous variable. Each dot in the bubble plot represents an independent effect, with dot size reflecting the weight of the combined main effect. As effect values increase, dot color deepens. The solid black line denotes the regression line; the gray area indicates the 95% confidence interval; the purple area represents the 95% prediction interval. The yellow dashed line marks the −0.5 time period effect. AIC: Akaike Information Criterion. BIC: Bayesian Information Criterion.

**Figure 8:**
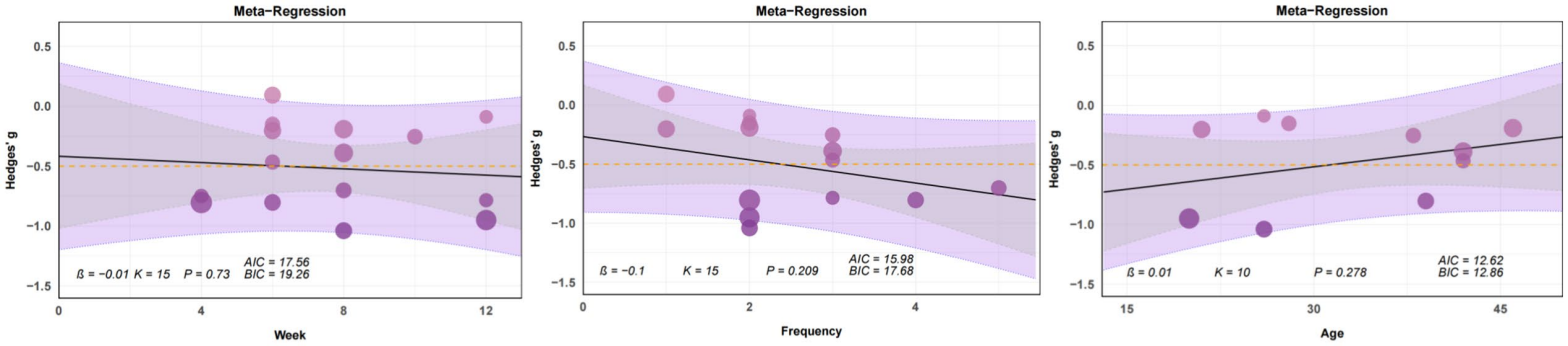
Plot of regression analysis results from the meta-analysis of yoga interventions to cope with anxiety (Week, Frequency, Age). This figure presents a bubble plot of linear regression analysis for anxiety indicators, illustrating the association effects of each continuous variable. Each dot in the bubble plot represents an independent effect, with dot size reflecting the weight of the combined main effect. As effect values increase, dot color deepens. The solid black line denotes the regression line; the gray area indicates the 95% confidence interval; the purple area represents the 95% prediction interval. The yellow dashed line marks the −0.5 time period effect. AIC: Akaike information criterion. BIC: Bayesian information criterion.

**Figure 9:**
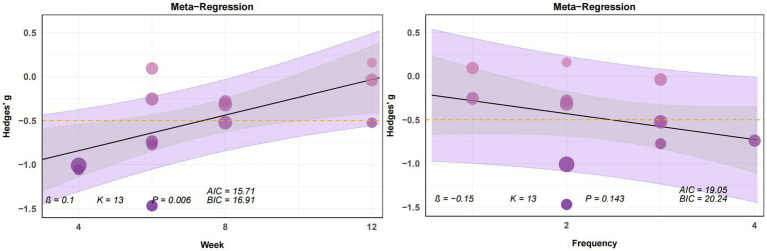
Plot of regression analysis results from the meta-analysis of yoga interventions to cope with depression (week, frequency). This figure presents a bubble plot of linear regression analysis for anxiety indicators, illustrating the association effects of each continuous variable. Each dot in the bubble plot represents an independent effect, with dot size reflecting the weight of the combined main effect. As effect values increase, dot color deepens. The solid black line denotes the regression line; the gray area indicates the 95% confidence interval; the purple area represents the 95% prediction interval. The yellow dashed line marks the -0.5 time period effect. AIC: Akaike Information Criterion. BIC: Bayesian Information Criterion. Given that only eight studies reported age data, we abandoned the meta-regression analysis examining the relationship between depression and age.

When analyzing the effects of yoga intervention on stress, we found that stress reduction exhibited a significant linear relationship only with age (*β* = −0.01, *p* < 0.05) (Figure 7). When examining the effects of yoga intervention on anxiety, we observed no significant linear relationships between anxiety reduction and intervention cycle, frequency, or age (Figure 8). In analyzing the effect of yoga intervention on depression, we found that depression showed a significant linear relationship only with intervention cycle (β = 0.1, *p* < 0.01) (Figure 9).

### Publication bias

**Figure 10:**
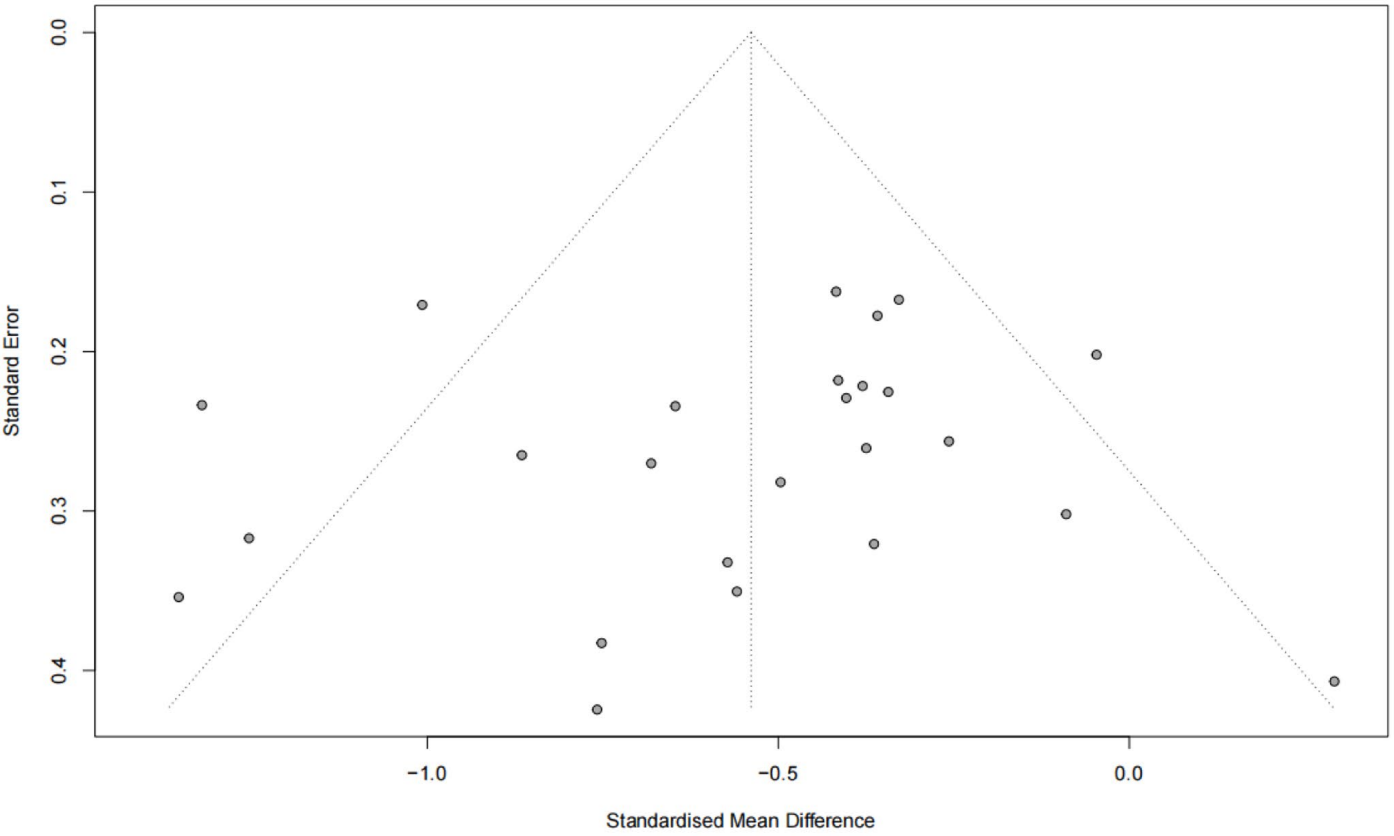
Publication bias funnel plot (stress).

**Figure 11:**
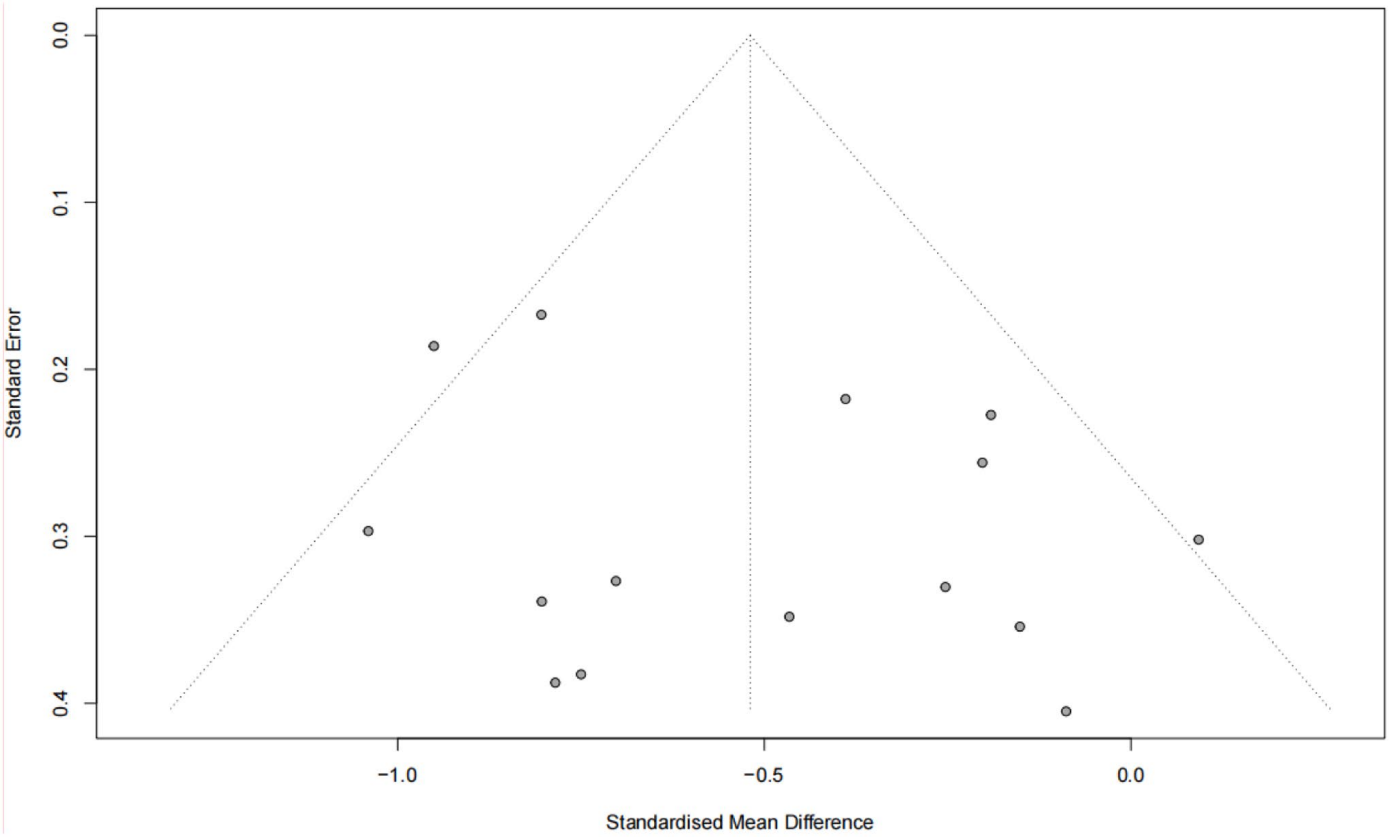
Publication bias funnel plot (anxiety).

**Figure 12:**
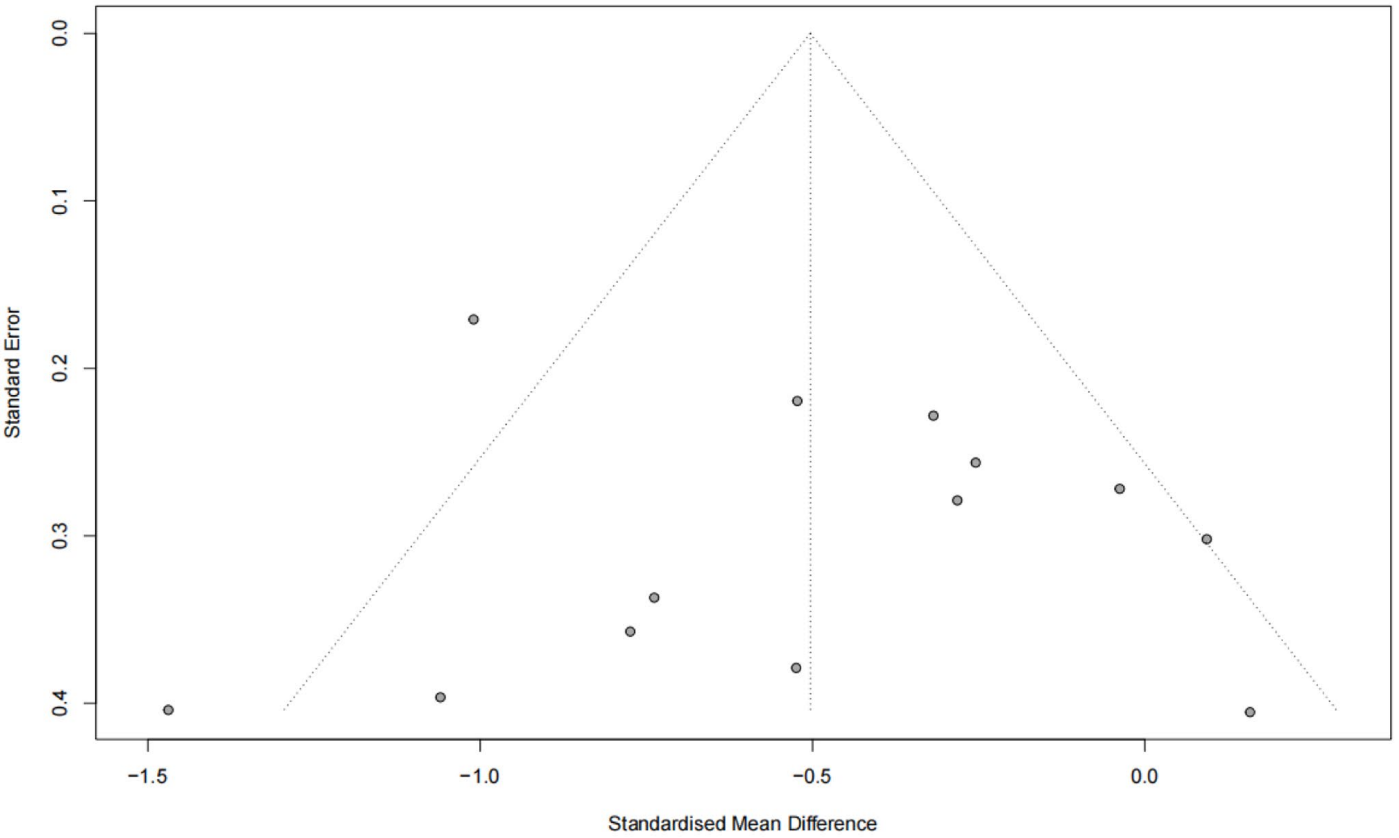
Publication bias funnel plot (depression).

The Egger test revealed no evidence of publication bias in the combined results for stress, anxiety, and depression (*p* > 0.05) (Figures 10–12).

### Certainty of evidence

The overall level of evidence for yoga interventions addressing stress, depression, and anxiety is low. Several factors contribute to this phenomenon: (1) High risk of bias across studies due to considerable uncertainty. (2) Participants, while not reporting significant psychological or physiological disorders, exhibited diversity in age range and occupational types. (3) Lack of measurement consistency, with outcomes for the same indicator derived from different scales. (4) Variations in yoga practice, as different yoga traditions may exert differing effects on outcomes (Supplementary Table 4).

## Discussion

### Summary of evidence

This systematic review examined 30 randomized controlled studies investigating yoga’s effects on emotions and stress. It further assessed the moderating effects of control group type, gender, age, intervention duration, frequency, single-session duration, and training supervision on outcomes. Final results indicate yoga effectively alleviates depression (low-level evidence), anxiety (low-level evidence), and stress (low-level evidence). Subgroup analyses revealed that different control groups significantly moderated effects on stress, depression, and anxiety. Age emerged as a significant moderator for stress, while intervention duration and single-session duration significantly moderated depression outcomes. Regression analysis indicated that yoga’s stress-relieving effects increased with age, whereas depression levels showed an upward trend as intervention duration lengthened.

### Effect of yoga on stress

The findings of this study align with those of other meta-analyses (Schleinzer et al., 2024; Martis et al., 2023; Erkin and Kocaçal, 2024; Schleinzer et al., 2024) covering populations such as healthcare professionals, students, and adults. These studies also identified positive effects of yoga in reducing stress, though only modest effects were observed in healthy individuals. The effect size obtained by this study is slightly higher than that of Rhodes’s (Rhoads et al., 2024) research (this study: 0.54 vs. Rhodes: 0.48). Rhoads et al. (2024) conducted a moderation analysis and found that longer practice duration was not statistically significant. This study reached the same conclusion through regression analyses of intervention duration and frequency. Yoga primarily involves stretching, breathing exercises, and body control, with activity intensity far below that of traditional resistance training or moderate-to-high-intensity aerobic exercise. Considering the physiological benefits of mechanical loading on the human body, changing the observation method is particularly important. Using only intervention duration and frequency as moderating factors is somewhat limited.

This study found that age is a significant moderator of yoga’s effect on stress. We conducted subgroup analyses and regression analyses. Subgroup analysis revealed that the SMD for participants aged 30 and above was twice that of those aged 30 and below, with I^2^ values of 0. Furthermore, regression analysis demonstrated that the model was statistically significant, proving that yoga intervention becomes more effective at alleviating stress as age increases.

### Effect of yoga on depression

Current research on using yoga exercises to improve depression has primarily focused on four groups: pregnant women (Ng et al., 2019), cancer patients (Ma et al., 2025), individuals with depression (Miao et al., 2023), and those with post-traumatic stress disorder (Nejadghaderi et al., 2024). All these populations experienced reduced depressive symptoms following yoga practice. The pooled effect size obtained in this study reached −0.50. These findings demonstrate that yoga exercises exert a significant effect in alleviating depression.

In subgroup analyses, we found that intervention duration and single-session length were significant moderating factors influencing depression. For subgroup analysis, we categorized intervention duration into two groups: 8 weeks or less and over 8 weeks. This division was made because only 3 out of 13 studies had intervention durations exceeding 8 weeks, and the heterogeneity for the 8-week or less group reached 59%. Therefore, caution is warranted when interpreting these results. Regarding single-session duration, 8 studies exceeded 50 min with heterogeneity below 25%, while only 5 studies lasted 50 min or less, exhibiting 35% heterogeneity. Although single sessions of 50 min or less demonstrated a large effect size for reducing depression, the moderate heterogeneity warrants cautious interpretation of these findings.

Rivera-Bonet et al. (2021) found a correlation between increased cortisol and depression, the regional volume of the hippocampus as well as brain-derived neurotrophic factor can map the level of depression in the human body, and studies have shown that long-term cortisol secretion causes regional atrophy in the hippocampus (Malykhin et al., 2025), and the secretion of brain-derived neurotrophic factor can affect neural pathways to prevent the increase in depression (Fan et al., 2024). Zhao et al. (2022) found in his study that the increase of brain-derived neurotrophic factor is affected by the intensity of exercise but not by the duration of exercise, although the increase of exercise intensity will fluctuate cortisol within a certain range, but for the release of BDNF play a good role in regulating the release of BDNF, due to the characteristics of the low intensity of traditional yoga exercise cannot be adequately stimulated to the release of BDNF, where the focus is on the exercise program arrangement.

### Effect of yoga on anxiety

This study included 15 literature reviews examining yoga interventions for anxiety management, yielding a moderate effect size (0.52). Subgroup and regression analyses revealed that intervention duration, frequency, and single-session length did not significantly moderate the effects. Sensitivity analysis reduced heterogeneity to 30%, but also correspondingly lowered the effect size. Overall, the evidence from this study indicates that yoga exercise demonstrates a small effect on anxiety reduction.

Previous studies have shown that individuals with high anxiety exhibit increased sympathetic nervous system activity and decreased parasympathetic nervous system activity (Bian et al., 2022). Due to the emphasis on breathing in yoga practice, parasympathetic activity is enhanced (Eda et al., 2020). Moreover, changes in anxiety levels are strongly correlated with neurotransmitter activity (Long et al., 2024). Yoga exercise has been shown to help maintain neurological homeostasis and stabilize neurotransmitter levels, while also influencing limbic system function as well as endocrine responses (Padmavathi et al., 2023). Based on findings from multiple studies, yoga practice appears effective in promoting physiological adaptations and reducing anxiety-related manifestations.

### Mechanism of action

Current research supports the positive effects of yoga on emotions and stress, and studies have explored the underlying mechanisms involved.

In terms of stress, an early study incorporating five literature sources examined the core mechanisms by which yoga alleviates stress. The findings indicate that positive affect, self-compassion, inhibition of the posterior hypothalamus, and salivary cortisol levels may mediate the relationship between yoga and stress (Riley and Park, 2015). In subsequent mechanism studies, Park recruited 144 participants for a yoga intervention and measured psychological changes. The findings revealed that all psychological resources (mindfulness, body consciousness, self-transcendence, spiritual peace, and social connectedness) were enhanced following the intervention, and these improvements were closely associated with emotional well-being (Park et al., 2020). Building on this foundation, another clinical study by Park measured 42 participants and found that yoga’s stress-relieving effects likely operate through five psychological mechanisms (increased mindfulness, interoceptive awareness, spiritual well-being, self-compassion, and self-control) by leveraging stress reactivity and integrating stress reduction mechanisms (Park et al., 2021).

Regarding depression, Naveen et al. (2013) research found that yoga intervention alone was more effective than antidepressant medication in alleviating depressive symptoms. Within the yoga-only intervention group, the reduction in depression levels showed a significant positive correlation with increased serum BDNF levels. Shapiro et al. (2007) study investigated the effects of yoga intervention on HRV. Results indicated that yoga intervention can improve depressive symptoms by regulating autonomic nervous system balance, but its efficacy is significantly modulated by individual baseline vagal tone. This also implies that intervention content must be tailored for individuals with different baseline conditions.

Regarding anxiety, Boni et al. (2018) conducted a cross-sectional study, which revealed that avoidance and mindfulness effectively mediated the relationship between yoga intervention and anxiety. Parkinson and Smith (2023) examined the benefits of yoga practice duration on anxiety through a cross-sectional study. Results indicated that interoceptive awareness, spiritual intelligence, mindfulness, and self-compassion each mediated the relationship between yoga experience and emotional dysregulation. Furthermore, emotional dysregulation mediated the relationship between yoga experience and depression, anxiety, and stress. In a study examining yoga experiences, Franklin et al. (2018) collected data on the experiences of 186 individuals during yoga practice and found that the experience of asanas was negatively correlated with anxiety symptoms and depression levels.

### Limitations

When reviewing this study, several limitations should be considered. During subgroup analysis, insufficient statistical power existed in certain subgroups due to the small number of included studies, making it impossible to completely rule out potential effects from moderating factors. Furthermore, given the limited number of included studies, concerns about model overfitting instability and inflated Type I error rates warrant cautious interpretation of the results.

Secondly, in the regression analysis, we were unable to convert the exercise intensity of yoga into metabolic equivalents (METs) for analysis. Relying solely on the intervention cycle, frequency, and duration per session may not fully reflect the cumulative effects of the intervention.

Third, this meta-analysis only selected English literature in the literature search and inclusion process, and only collected peer-reviewed published literature, but not gray literature. Finally, some of the literature was not included in the meta-analysis due to the lack of open source and authors could not be contacted, which may have some influence on the final results, so please take the results of this study with caution.

## Conclusion

Yoga practice has been proven effective in reducing stress and alleviating symptoms of depression and anxiety. As age increases, yoga interventions yield greater effectiveness in stress reduction. Future considerations should include incorporating yoga as an adjunct therapy within mental health prevention strategies.

### Suggest

Reducing depression, anxiety and stress plays a crucial role in preventing various chronic diseases (Fan et al., 2024; Burke et al., 2018; Dong et al., 2020) and risky behaviors (He et al., 2025). Currently, yoga exercise has been proved to be a convenient and effective means of health care and rehabilitation, which can be done on the basis of physical exercise as well as psychological adjustment.

The results obtained in this study support the use of yoga as a non-pharmacological intervention for emotional behavior modification and stress relief, but with a critical approach, this study makes some pertinent recommendations:

1. We recommend yoga intervention as a strategy for alleviating emotional distress and reducing stress. Through subgroup analysis and regression analysis, we obtained reliable evidence indicating that yoga intervention yields greater stress-reducing effects with increasing age.
2. Considering that intervention duration, frequency, and single-session duration do not adequately reflect the overall intervention effect, we suggest quantifying the comprehensive intervention effect in future meta-analyses using METs.
3. Given the limited sample sizes in intervention studies, future meta-analyses may adopt Hedges’ g effect size calculation for effect consolidation. This method corrects for small-sample bias, preventing overestimation of results.
4. Traditional yoga emphasizes low intensity, which stabilizes cortisol secretion but fails to sufficiently stimulate neurotrophic factor release. This study recommends adjusting yoga exercise intensity to avoid purely low-intensity scenarios, thereby achieving physiological adaptation effects.
5. Given that existing research has confirmed the relationship between yoga postures and emotions during yoga interventions, yoga designers should consider the effects produced by postures when developing their programs.

## Data Availability

The original contributions presented in the study are included in the article/Supplementary material, further inquiries can be directed to the corresponding authors.
