# Supplementary material for: The effects of yoga exercise on stress relief capacity and emotional changes: a systematic review and meta-analysis
Source: Front Psychol. 2026 Feb 26;17:1707131. doi: 10.3389/fpsyg.2026.1707131 (PMC12980539; doi:10.3389/fpsyg.2026.1707131)
Supplement: Supplementary file 1 [file Supplementary_file_1.pdf]

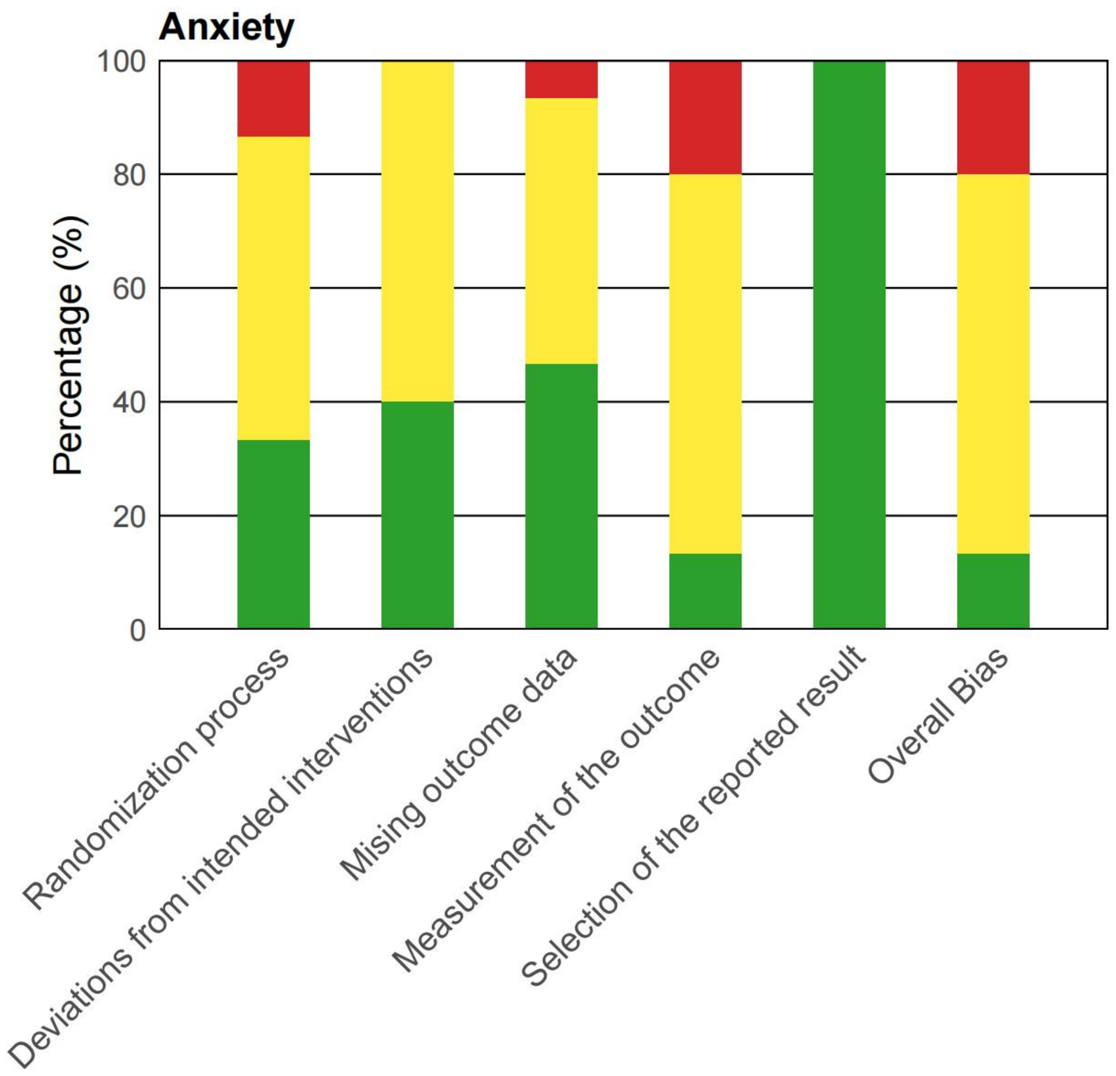

Supplementary Fig. 1 Risk of Bias (Anxiety)

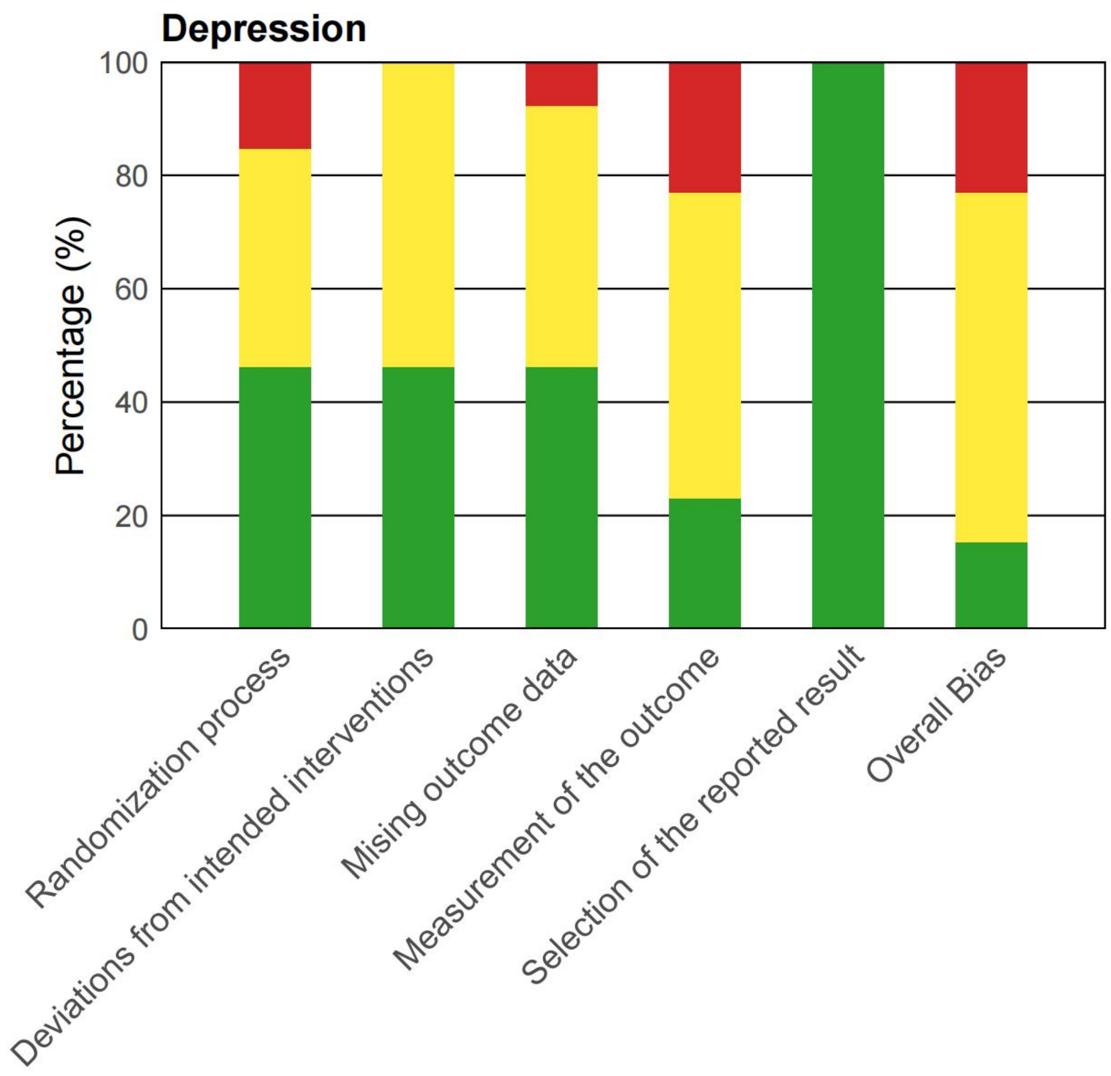

Supplementary Fig. 2 Risk of Bias (Depression)

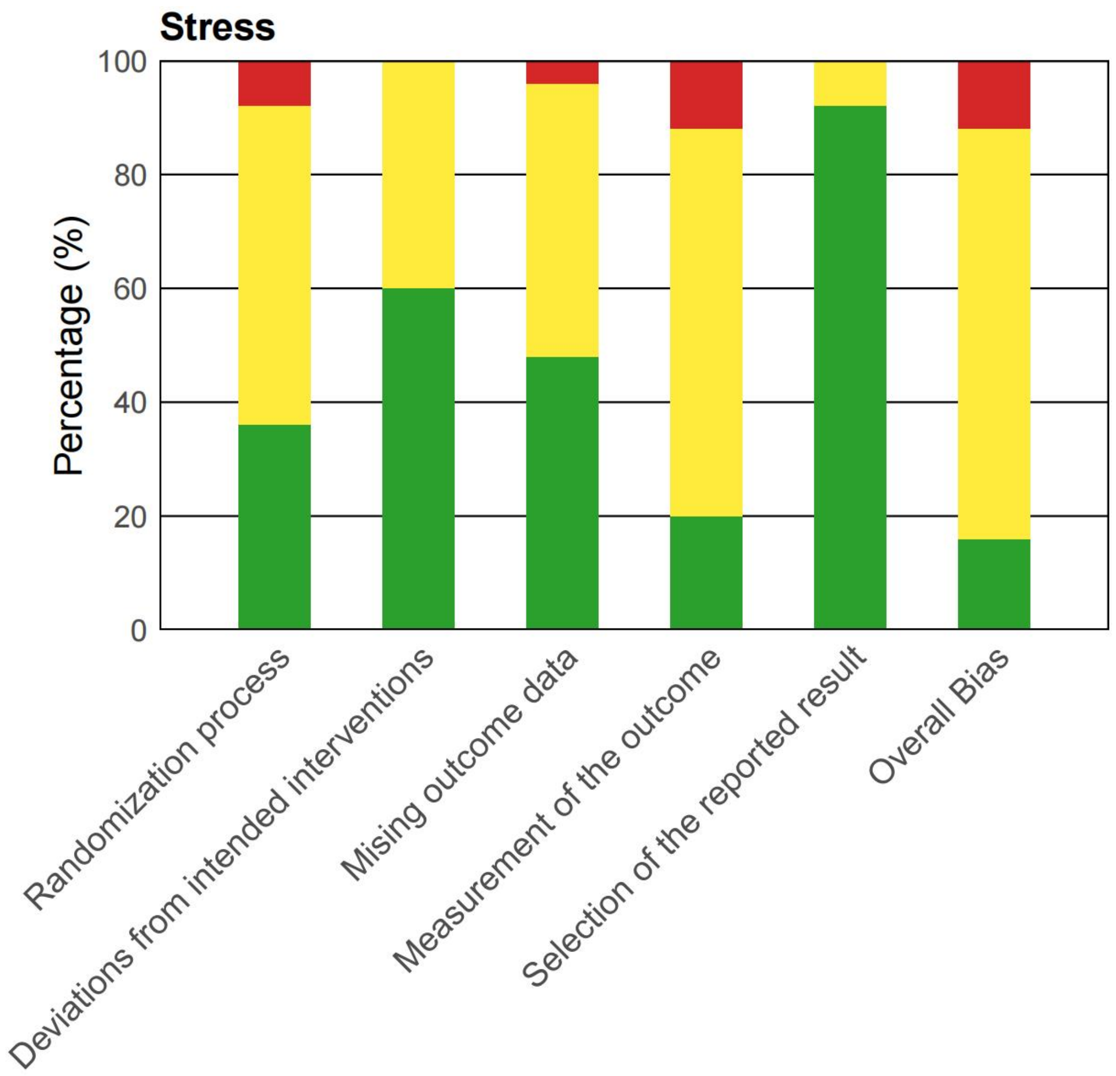

Supplementary Fig. 3 Risk of Bias (Stress)

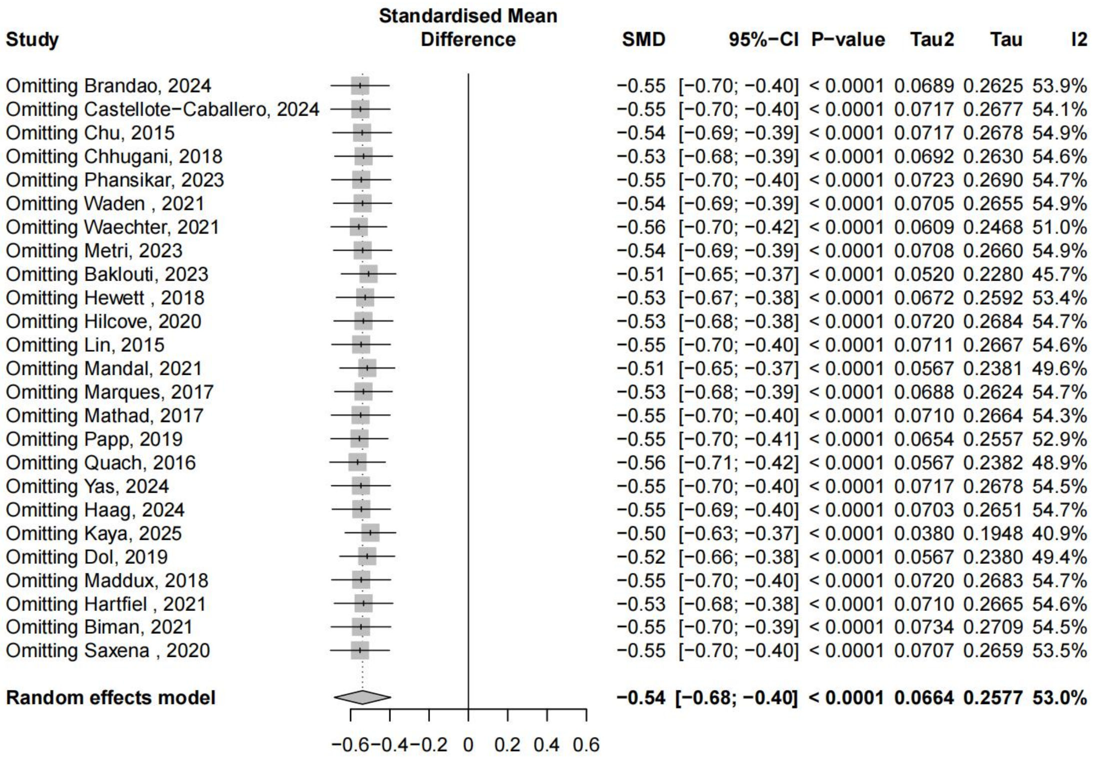

Supplementary Fig. 4 Sensitivity Analysis (Stress)

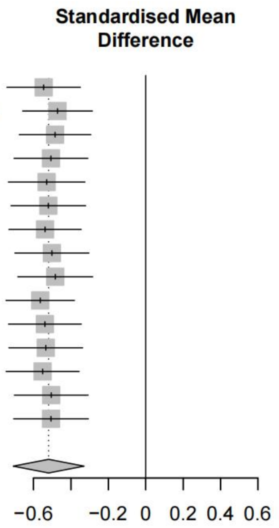

Study	SMD	95%−CI	P−value	Tau2	Tau	I2
Omitting Brandao, 2024	−0.55	[−0.74; −0.35]	< 0.0001	0.0557	0.2360	40.6%
Omitting Castellote−Caballero, 2024	−0.47	[−0.66; −0.29]	< 0.0001	0.0394	0.1984	30.2%
Omitting Chu, 2015	−0.48	[−0.68; −0.29]	< 0.0001	0.0508	0.2254	38.3%
Omitting Chhugani, 2018	−0.51	[−0.70; −0.31]	< 0.0001	0.0613	0.2476	45.0%
Omitting Phansikar, 2023	−0.53	[−0.73; −0.32]	< 0.0001	0.0650	0.2550	44.2%
Omitting Waden , 2021	−0.52	[−0.72; −0.32]	< 0.0001	0.0629	0.2508	45.5%
Omitting Waechter, 2021	−0.54	[−0.73; −0.34]	< 0.0001	0.0561	0.2369	42.4%
Omitting Metri, 2023	−0.50	[−0.70; −0.30]	< 0.0001	0.0608	0.2466	44.3%
Omitting Baklouti, 2023	−0.48	[−0.68; −0.28]	< 0.0001	0.0559	0.2365	38.7%
Omitting Papp, 2019	−0.56	[−0.75; −0.38]	< 0.0001	0.0411	0.2027	31.9%
Omitting Gunebakan, 2023	−0.54	[−0.73; −0.34]	< 0.0001	0.0568	0.2383	42.4%
Omitting Cheema, 2013	−0.53	[−0.73; −0.34]	< 0.0001	0.0595	0.2439	43.6%
Omitting Maddux, 2018	−0.55	[−0.75; −0.36]	< 0.0001	0.0528	0.2299	38.4%
Omitting Varambally, 2013	−0.51	[−0.70; −0.31]	< 0.0001	0.0608	0.2466	44.8%
Omitting Devi, 2024	−0.51	[−0.71; −0.31]	< 0.0001	0.0627	0.2504	45.2%
**Random effects model**	**−0.52**	**[−0.71; −0.33]**	**< 0.0001**	**0.0563**	**0.2372**	**41.5%**

Supplementary Fig. 5 Sensitivity Analysis (Anxiety)

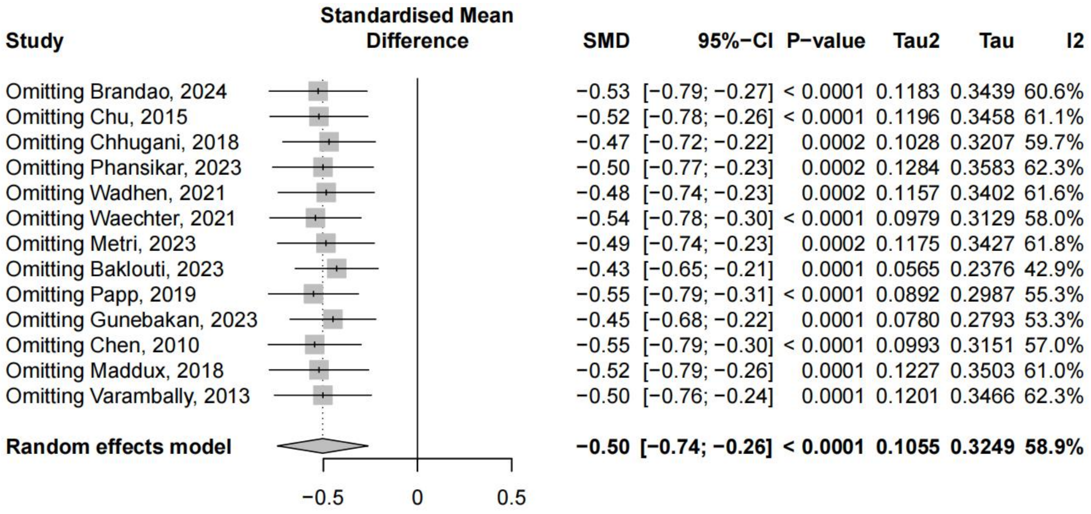

Supplementary Fig. 6 Sensitivity Analysis (Depression)

Supplementary Table 1 Subgroup Analysis Results (Stress)

Subgroup	K(N)	Hedges'g	95%CI	Tau^2	Tau	Q	I^2	P_d value
1.Control type								
Active Control Group	4	-0.184	(-0.470;0.102)	0	0	2.08	0.0%	
Control Group	11	-0.670	(-0.899;-0.440)	0.085	0.292	25.67	61.0%	0.033*
Waitlist Group	10	-0.512	(-0.707;-0.317)	0.036	0.189	14.50	37.9%	
2.Gender								
Mixed	20	-0.539	(-0.710;-0.368)	0.090	0.300	49.69	61.8%	0.868
Female	5	-0.512	(-0.780;-0.245)	0	0	1.32	0.0%	
3.Age								
Under 30 years old	8	-0.281	(-0.438;-0.124)	0	0	4.46	0.0%	0.009**
Over 30 years old	11	-0.568	(-0.718;-0.419)	0	0	8.85	0.0%	
3.Intervention Cycle								
8 weeks and under	17	-0.560	(-0.742;-0.378)	0.077	0.277	36.3	55.9%	0.634
More than 8 weeks	8	-0.489	(-0.715;-0.263)	0.044	0.209	13.64	48.7%	
4.Intervention frequency								
3 times or fewer	19	-0.529	(-0.715;-0.344)	0.099	0.315	47.64	62.2%	0.951
More than 3 times	6	-0.538	(-0.728;-0.347)	0	0	3.36	0.0%	
5.Intervention time								
Over 50 minutes	13	-0.403	(-0.541;-0.264)	0	0.001	12.91	7.1%	0.106
50 minutes or less	10	-0.654	(-0.927;-0.381)	0.129	0.360	31.49	71.4%	
6.Training Monitoring								
Online	5	-0.609	(-0.90;-0.317)	0.053	0.231	7.96	49.7%	
Offline	14	-0.626	(-0.846;-0.406)	0.098	0.313	31.45	58.7%	0.262
Offline+Online	3	-0.256	(-0.655;0.142)	0.059	0.244	3.83	47.8%	

Supplementary Table 2 Subgroup Analysis Results (Anxiety)

Subgroup	K	Hedges'g	95%CI	Tau^2	Tau	Q	I^2	P_d value
1.Control type								
Active Control Group	3	-0.081	(-0.425;0.263)	0	0	0.56	0.0%	
Control Group	7	-0.765	(-0.951;-0.579)	<0.0001	0.002	7.36	18.5%	0.001**
Waitlist Group	5	-0.419	(-0.668;-0.171)	0	0	2.68	0.0%	
2.Gender								
Mixed	11	-0.465	(-0.683;-0.246)	0.061	0.247	18.94	47.2%	0.270
Female	4	-0.711	(-1.091;-0.331)	0.034	0.186	3.81	21.2%	
3.Age								
Under 30 years old	5	-0.537	(-0.954;-0.121)	0.139	0.373	11.28	64.5%	0.510
Over 30 years old	5	-0.376	(-0.616;-0.136)	0	0	2.46	0.0%	
3.Intervention Cycle								
8 weeks and under	11	-0.489	(-0.701;-0.277)	0.049	0.221	16.50	39.4%	0.680
More than 8 weeks	4	-0.589	(-1.021;-0.158)	0.094	0.306	5.97	49.8%	
4.Intervention frequency								
3 times or fewer	12	-0.470	(-0.690;-0.249)	0.074	0.272	22.77	51.7%	0.222
More than 3 times	3	-0.750	(-1.143;-0.357)	0	0	0.05	0.0%	
5.Intervention time								
Over 50 minutes	9	-0.523	(-0.807;-0.239)	0.100	0.317	18.57	56.9%	0.982
50 minutes or less	6	-0.527	(-0.776;-0.279)	0.021	0.145	5.35	6.5%	
6.Training Monitoring								
Online	5	-0.461	(-0.740;-0.182)	0.037	0.193	5.88	32.0%	
Offline	8	-0.621	(-0.892;-0.350)	0.062	0.250	12.08	42.1%	0.632
Offline+Online	2	-0.312	(-1.170;0.544)	0.264	0.514	3.19	68.6%	

Supplementary Table 3 Subgroup Analysis Results (Depression)

Subgroup	K	Hedges'g	95%CI	Tau^2	Tau	Q	I^2	P_d value
1.Control type								
Active Control Group	3	-0.058	-0.403;0.286	0	0	1.12	0.0%	
Control Group	4	-0.845	-1.288;-0.402	0.120	0.346	7.47	59.8%	0.019*
Waitlist Group	6	-0.457	-0.692;-0.222	0.004	0.069	5.97	16.3%	
2.Gender								
Mixed	9	-0.391	-0.663;-0.120	0.094	0.306	20.36	60.7%	0.134
Female	4	-0.831	-1.337;-0.324	0.143	0.379	6.60	54.5%	
3.Age								
Under 30 years old	4	-0.440	-1.057; 0.177	0.285	0.533	9.40	68.1%	0.811
Over 30 years old	4	-0.521	-0.782;-0.261	0	0	1.71	0.0%	
3.Intervention Cycle								
8 weeks and under	10	-0.596	-0.863; -0.330	0.102	0.319	22.17	59.4%	0.044*
More than 8 weeks	3	-0.120	-0.500;0.259	0	0	1.70	0.0%	
4.Intervention frequency								
3 times or fewer	11	-0.447	-0.711; -0.184	0.114	0.337	26.82	62.7%	0.142
More than 3 times	2	-0.873	-1.376;-0.369	0	0	0.38	0.0%	
5.Intervention time								
Over 50 minutes	8	-0.272	-0.476;-0.069	<0.0001	0.001	9.24	24.3%	0.002*
50 minutes or less	5	-0.836	-1.136;-0.536	0.038	0.197	6.23	35.8%	
6.Training Monitoring								
Online	5	-0.761	-1.130;-0.393	0.102	0.320	10.28	61.1%	
Offline	6	-0.344	-0.614;-0.074	0.021	0.145	7.49	33.3%	0.125
Offline+Online	2	-0.173	-0.773; 0.426	0.073	0.271	1.63	38.5%	

Supplementary Table 4 GRADE Evidence Level Results

Outcome	participant	Level of evidence					Hedges’g（95%*CI*）	level
		risk of bias	inconsistency	indirectness	inaccuracy	publication bias		
Stress	1838（25RCT）	serious	serious	serious	not serious	not serious	-0.54 (-0.68,-0.40)	low
Depression	728（13RCT）	serious	serious	serious	not serious	not serious	-0.50 (-0.74,-0.26)	low
Anxiety	879（15RCT）	serious	serious	serious	not serious	not serious	-0.52 (-0.71,-0.33)	low

GRADE quality of clinical evidence and grade of recommendation:

High: The group has full confidence in the results

Medium: Subjects are moderately confident of the outcome

Low: Subjects have limited confidence in the results

Very low: subject group has very limited confidence in the results

Note: RCT: randomized controlled study; Hedges'g: combined
